# Neutrophil extracellular traps in tumor progression of gynecologic cancers

**DOI:** 10.3389/fimmu.2024.1421889

**Published:** 2024-11-01

**Authors:** Hong Chen, Ying Zhou, Yaling Tang, Jianfa Lan, Chao Lin, Qionghua Chen, Hongying Kuang

**Affiliations:** ^1^ Department of Obstetrics and Gynecology, The First Affiliated Hospital of Xiamen University, Xiamen, China; ^2^ The Second Department of Gynecology, The First Affiliated Hospital, Heilongjiang University of Chinese Medicine, Harbin, China

**Keywords:** tumor immune microenvironment, neutrophils, neutrophil extracellular traps, metastasis and recurrence, gynecologic cancers, new treatment strategy

## Abstract

This article delves into the intricate interplay between tumors, particularly gynecologic malignancies, and neutrophil extracellular traps (NETs). The relationship between tumors, specifically gynecologic malignancies, and NETs is a multifaceted and pivotal area of study. Neutrophils, pivotal components of the immune system, are tasked with combating foreign invaders. NETs, intricate structures released by neutrophils, play a vital role in combating systemic infections but also play a role in non-infectious conditions such as inflammation, autoimmune diseases, and cancer. Cancer cells have the ability to attract neutrophils, creating tumor-associated neutrophils, which then stimulate the release of NETs into the tumor microenvironment. The impact of NETs within the tumor microenvironment is profound and intricate. They play a significant role in influencing cancer development and metastasis, as well as modulating tumor immune responses. Through the release of proteases and pro-inflammatory cytokines, NETs directly alter the behavior of tumor cells, increasing invasiveness and metastatic potential. Additionally, NETs can trigger epithelial-mesenchymal transition in tumor cells, a process associated with increased invasion and metastasis. The interaction between tumors and NETs is particularly critical in gynecologic malignancies such as ovarian, cervical, and endometrial cancer. Understanding the mechanisms through which NETs operate in these tumors can offer valuable insights for the development of targeted therapeutic interventions. Researchers are actively working towards harnessing this interaction to impede tumor progression and metastasis, opening up new avenues for future treatment modalities. As our understanding of the interplay between tumors and NETs deepens, it is anticipated that novel treatment strategies will emerge, potentially leading to improved outcomes for patients with gynecologic malignancies. This article provides a comprehensive overview of the latest research findings on the interaction between NETs and cancer, particularly in gynecologic tumors, serving as a valuable resource for future exploration in this field.

## 1 Introduction

Cancer is a kind of complex systemic disease, which is the result of a variety of factors (1, 2). Its formation and development are not only related to the random variation of the body’s parenchymal cell genome, but also the disorder of the host immune microenvironment and the decline of immune surveillance ability play an important role in this process (1–3). The interaction between tumor cells and immune cells is also an important factor affecting tumor progression (1, 4, 5). The tumor immune microenvironment (TIME) is influenced by different chemokines and cytokines and is orchestrated by immune and tumor cells interacting (5–7). It is essential to the development, spread, invasion, and metastasis of malignancies. Immune cells invading tumors have the ability to either stimulate or prevent tumorigenesis. However, in the occurrence and development of tumor, which is a chronic inflammation, neutrophils also become an important force leading to the formation and progression of tumor, which is an “incurable wound” (8–10).

Tumor cells are a type of abnormal cells with immunogenicity (11, 12). In a state of maximum immunological vigilance, the immune system is able to promptly identify and eradicate aberrant transformed cells, thus preventing the occurrence of tumors (12). The microenvironment’s inflammatory mediators have the ability to gather in additional neutrophils and contribute to the microenvironment (13). The hallmarks of inflammation that are linked to cancer are neutrophil recruitment and activation (14, 15). Tumor-associated neutrophils(TANs) are neutrophils that settle down or infiltrate the tumor microenvironment (15). T lymphocytes play an important role in the first stage of tumor immune editing “immune surveillance” (16). Interferon (IFN)-g-secreting cytotoxic CD8+ T lymphocytes (CTLs) are a major constituent of the TIME and constitute a uniform population of cytotoxic cells. CTLs promote anti-tumor responses and enhance prognosis for patients (17). During the aging process, naive T cells decrease, memory and effector T cells increase, the diversity and sensitivity of T cell receptors decrease, and the immune surveillance ability is weakened (17). Regulatory T cells (Tregs) foster tumor cells and evade the immune system by attenuating immunological responses. Th cells develop distinct populations with varying functions, including Th1, Th2, and Th17 cells (18). Th1 cells stimulate CTLs, influence anti-tumor immunity, and are linked to better prognoses. Conversely, Th2 cells limit Th1 responses, support humoral immunity, and are linked to a worse prognosis. Th17 cells show heterogeneity in human cancer, expressing diverse cytokines, transcriptional factors, and activated markers, which affects patient prognosis (18). Combined with increased exposure to external tumorigenic factors, the genomic instability of cells increases. Tumor cells with low-immunogenic cannot be eliminated and are in a dormant state, which are temporarily in the state of “immune equilibrium”. When a certain stress event occurs and becomes the trigger, inflammation may break out in the body, and the immunosuppressive microenvironment created and manufactured by typical immune cells can promote tumor progression, thus entering the third stage of immune editing, “immune escape” (19, 20).

The initial line of defense against different types of infections and malignancies is the neutrophil (21). They primarily function as innate effector cells and account for over 70% of human circulating leukocytes. Neutrophils, the immune cell subtype with the greatest number of cells, are key players in acute infection and tissue damage (22, 23). They additionally prove the most beneficial for wound healing, which is due to their ability to degranulate, phagocytose, and form neutrophil extracellular traps (NETs) (1, 10).

**Figure 1:**
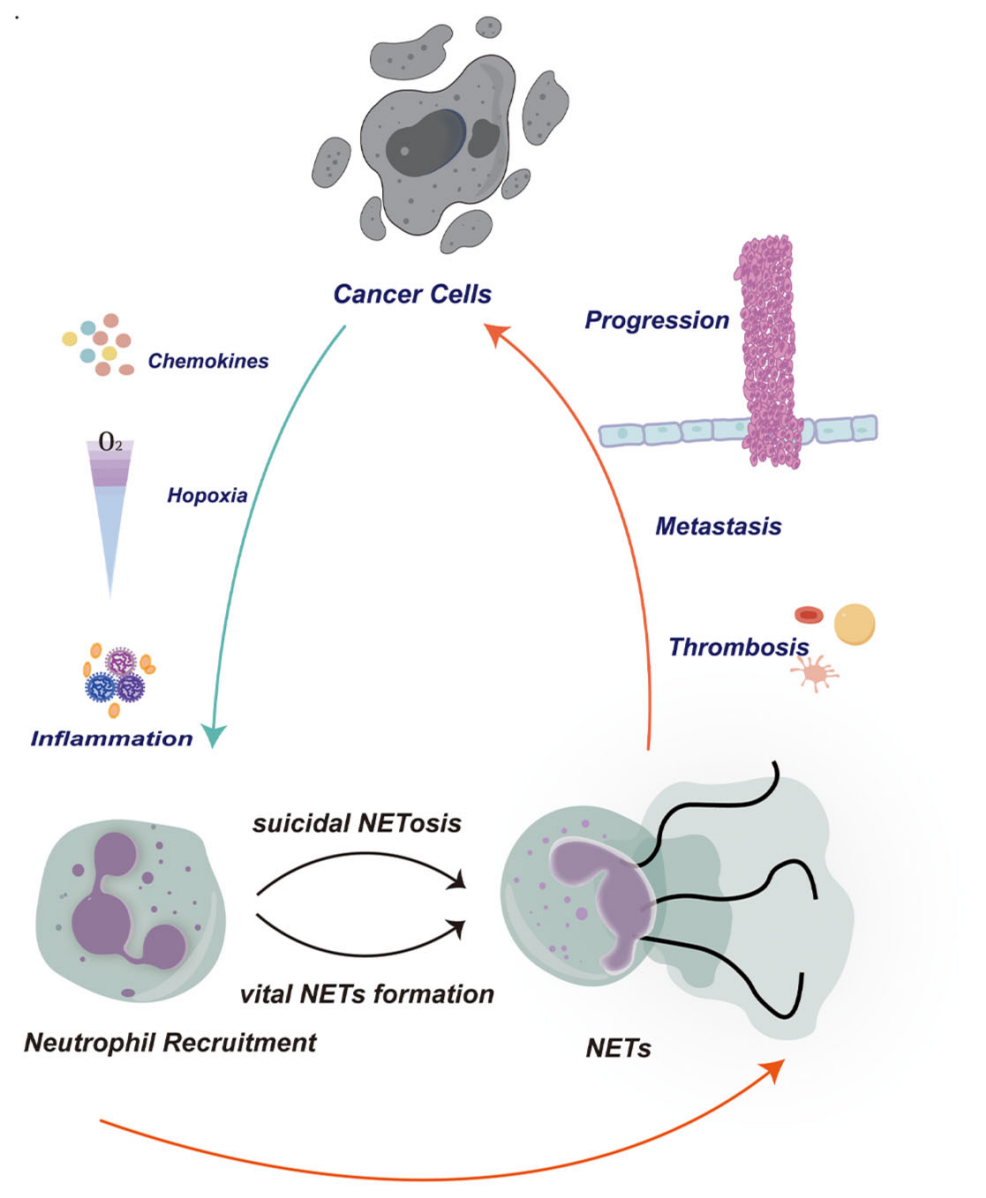
In the tumor microenvironment, cancer cells can release signaling molecules such as chemokines and cytokines to recruit neutrophils to the surrounding tissue of the tumor. These signaling molecules can attract neutrophils to migrate towards the tumor through various pathways. These neutrophils are activated and release neutrophil extracellular traps (NETs). The release of NETs can promote the migration and invasion of tumor cells, while also inducing the formation of blood clots to support the translocation of tumor cells. This phenomenon plays an important role in the tumor microenvironment, accelerating the progression of tumors by altering the tumor microenvironment, regulating immune responses, and influencing angiogenesis mechanisms. Therefore, the interaction between cancer cells, neutrophils, and NETs plays a crucial role in the progression and metastasis of tumors. A deeper understanding of this cooperative relationship can help us better comprehend the complexity of the tumor microenvironment and develop new therapeutic strategies.

In particular, the structure of NETs —”flying frame”, produced by neutrophils has also been confirmed to be the material basis of inflammatory response and coagulation system (**Figure 1**). NETs were first identified as a nucleic acid-based structure highlighted in bacterial immunity in 2004 (24). Activated neutrophil-ejected net-like structures are formed out of decondensed extracellular chromatin filaments embellished with granular proteins, involving histones, which are frequently citrullinated, neutrophil elastase (NE), cathepsin G, myeloperoxidase (MPO), and matrix metalloproteinases 9 (MMP9) (25–28). In fact, according to recent discoveries, NETs have become essential for the emergence and progression of malignancies (26–29). The formation of pre-metastatic niches, the resurgence of dormant metastases, and the possibility for direct tumor growth via related proteases like NE and MMP9 via the proteolytic restructuring of laminin are all reliant on them (30–32). In order to promote the adherence, invasion, and migration of circulating tumor cells, NETs may additionally attach to them and serve as an adhesion substrate (28, 30, 32–35).

Insufficient information is known concerning the mutual interaction of NETs with invading immune cells in the TIME of gynecologic cancers, compared to the direct impact on cancer cells (29, 31). It is also not widely recognized how NETs, that encompass both innate and adaptive immunity, interact with TIME of gynecologic cancers. A more thorough understanding of the connection between the TIME and NETs might encourage more potent tactics. With the objective to gain insight into the synergies between NETs and TIME on progression of tumors, this review will bring together the body of knowledge currently accessible in this field.

## 2 The ways in which NETs form

Suicidal NETosis is and vital NET formation are the two major types of NET development (36, 37).

### 2.1 Suicidal NETosis

The onset of suicidal NETosis is initiated by the activation of neutrophils through various triggers, such as immunological complexes, specific autoantibodies, cholesterol or calcium salt crystals, or phorbol-12-myristate-13-acetate (PMA) (36, 38–40). This activation induces an elevation in cytosolic Ca2+ levels, which in turn activates the NADPH oxidase (NOX) complex, resulting in the generation of reactive oxygen species (ROS) via Raf/MEK/ERK signaling (41, 42). Subsequently, NOX and ROS complexes stimulate protein-arginine deiminase 4 (PAD4) to modify histones, facilitating the movement of NE and MPO from neutrophil granules to the nucleus, thereby causing chromatin decondensation. The decondensed chromatin is eventually released during cellular lysis, combining with granular and cytosolic proteins in the cytoplasm. Suicidal NETosis is characterized by the demise of a cell through membrane dissolution, a process that may span several hours to complete (41, 43, 44).

### 2.2 Vital NETs

Shortly after neutrophil activation, the formation of vital NETs occurs independently of cell death and without membrane rupture. Stimuli such as lipopolysaccharide (LPS) from gram-negative bacteria or S. aureus initiate this process through complement receptors (CR) and toll-like receptors (TLRs) (45, 46). The NOX complex and reactive oxygen species (ROS) are not required for the generation of vital NETs. The release of nuclear DNA during the crucial NET formation process is associated with three specific morphological changes: nuclear decondensation, disruption of the nuclear envelope, and expansion of the nuclear envelope leading to vesicle release (29, 36, 44–46). Infectious diseases tend to encourage a higher frequency of vital NET formation compared to non-infectious diseases, as evidenced by the continued survival of neutrophils with their capacity for antimicrobial activities such as phagocytosis, chemotaxis, and bacterial killing (46–48).

### 2.3 Mitochondrial DNA NETs

In addition to the NETs formed by the release of nuclear DNA, research has identified NETs composed of mitochondrial DNA. This indicates that cells can utilize both nuclear and mitochondrial DNA when responding to infection or damage (49–51). Research has demonstrated that, after pretreatment with granulocyte/macrophage colony-stimulating factor (GM-CSF), neutrophils can generate NETs upon short-term stimulation with Toll-like receptor 4 (TLR4) or complement factor 5a (C5a) receptors. These NETs, produced by cells, contain mitochondrial DNA but are devoid of nuclear DNA. Additionally, inhibition of reactive oxygen species (ROS) through pharmacological or genetic approaches reveals that NETs formation relies on ROS. Neutrophils activated by GM-CSF and C5a show higher survival rates compared to resting neutrophils, which do not produce NETs. Overall, mitochondrial DNA release and NET formation by neutrophils do not necessitate cell death or restrict cell lifespan (52). Another study investigated the role of mitochondrial DNA (mtDNA) in the formation of NETs following trauma and orthopedic surgery. The findings revealed that these NETs were solely composed of mtDNA, lacking nuclear DNA. They emerged as a response to injury and continued to exist post-surgery. These observations suggest that mtDNA-based NETs could act as an indicator of heightened immune activation and may impact the timing of surgical interventions to mitigate inflammatory complications (49).

### 2.4 Tumor cells cause NETs development

The detection of NETs in tumor tissues has been revealed in multiple instances (27–29, 31, 32, 34, 44). Electron microscopy demonstrated that neutrophils were destroyed and did not show signs of DNA-containing vesicles emerging from intact neutrophils, which is consistent with the co-cultivation of cancer cells with neutrophils leading to NETs formation within 3 hours (4, 34). All of these results pointed to the induction of suicidal rather than vital NETs production by cancer cells.

## 3 Mechanisms of NETs in cancer development and progression

**Figure 2:**
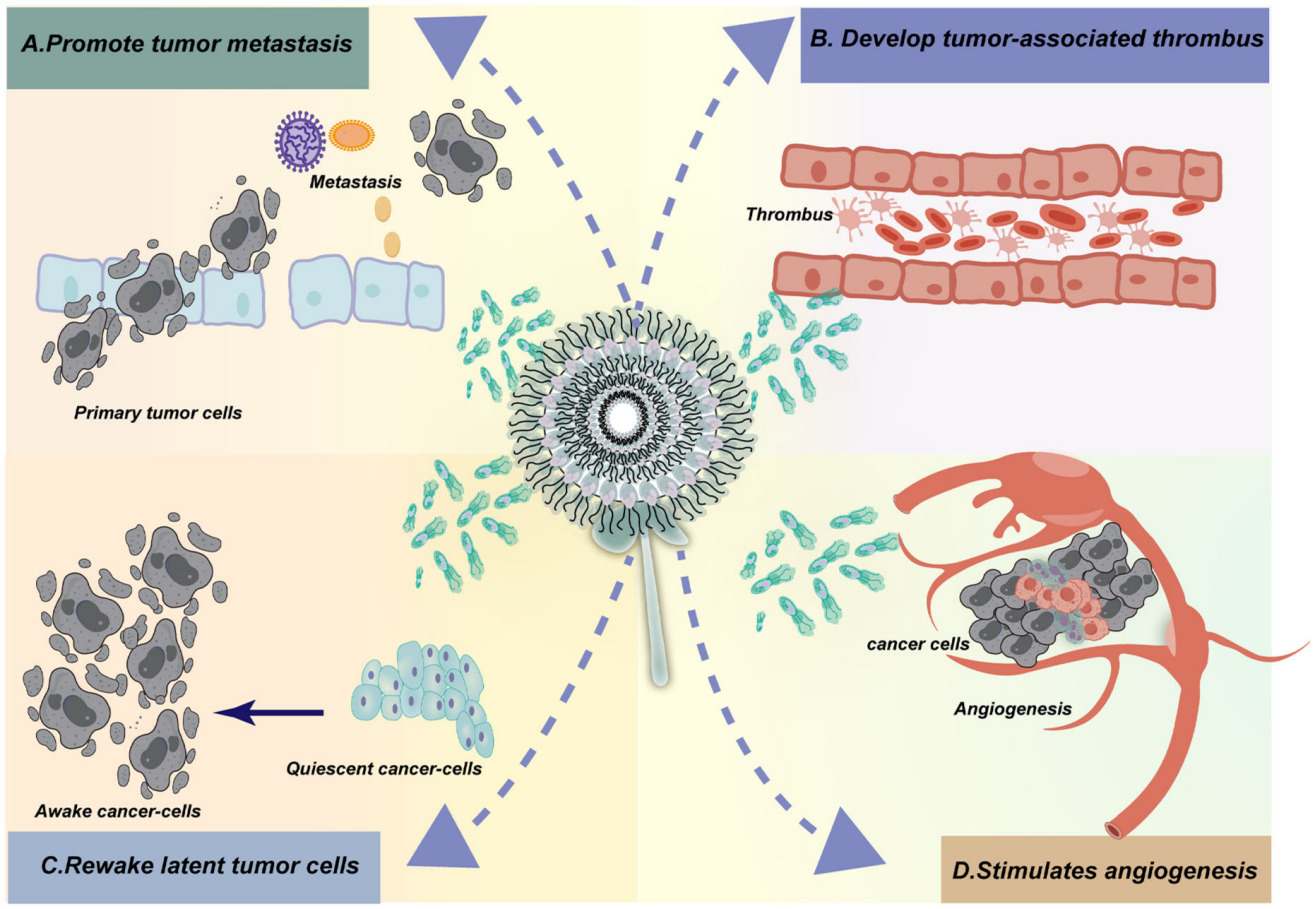
Neutrophil extracellular traps (NETs) have been implicated in cancer development and progression through various mechanisms. The activation of NETs by tumor cells has been shown to enhance the invasion and migration of tumors, thereby facilitating metastasis. Additionally, NETs released following surgery or under stress conditions have been implicated in stimulating tumor recurrence and metastasis by creating an inflammatory environment that supports cancer cell survival and proliferation. Furthermore, NETs aid in the survival of circulating tumor cells (CTCs) by promoting their adhesion and invasion into surrounding tissues. In the context of thrombosis, NETs composed of tissue proteases, cytokines, and DNA have been found to contribute to the development of venous and arterial thrombosis. Studies have shown that cancer patients have an increased risk of thrombosis, which can be attributed to the promotion of NET formation by tumor cells and platelets. Moreover, the release of NETs by neutrophils in different types of cancer has been associated with an elevated risk of thrombosis. Overall, NETs play a complex role in cancer development and progression, influencing various aspects of tumor biology including growth, angiogenesis, immune evasion, inflammation, and metastasis. Targeting NETs or their associated pathways may therefore represent a novel therapeutic strategy for cancer treatment.

In addition to promoting tumor formation by exacerbating chronic inflammation, neutrophils may also have a direct carcinogenic potential (10, 24). Recent studies have shown that in certain circumstances, neutrophils can directly participate in the formation and development of tumors (**Figure 2**). For example, neutrophils could directly promote tumor growth by releasing factors that stimulate tumor growth, promoting angiogenesis, and suppressing immune responses. Additionally, neutrophils may interact with tumor cells to promote tumor cell migration and invasion, thereby facilitating tumor spread and metastasis (10, 24, 25, 27, 28). Therefore, neutrophils may play a more complex role in tumor formation and development, beyond the mechanisms involving chronic inflammation. These new findings provide important clues for better understanding the interactions between different cell types in the tumor microenvironment and offer new research directions for future cancer treatment and prevention. Recently, in several chemical carcinogenesis mouse models, neutrophils have been shown to be crucial for tumor formation (29–32). In the past decade, the emergence of single-cell technologies has revealed significant heterogeneity in the state of neutrophils within tumors. It is now recognized that myeloid-derived cells, including neutrophils, are highly plastic cells in cancer, akin to the diverse reservoir of monocytes/macrophages. Some of this heterogeneity may be related to differences in the ability of neutrophils to migrate or form NETs, as previously described in different stages of the neutrophil lifecycle (21).

### 3.1 NETs promote tumor metastasis

#### 3.1.1 NETs can be activated by tumor cells which facilitates invasion and migration of tumors

The invasion and migration of tumors are enhanced by NETs (44, 53, 54). Certain breast cancer cells, such as those with significant G-CSF secretion abilities, have been shown *in vitro* to be able to activate neutrophils to make NETs, which can then be seen to promote the invasion and migration of the tumor cells and reverse their course. Additionally, pancreatic cancer cell lines produced by inducing NETs have improved invasion and migratory capabilities (55).

Polymorphonuclear myeloid-derived suppressor cells (PMN-MDSCs) play a crucial role in the tumor microenvironment, characterized by immune suppression that significantly reduces anti-tumor immune responses. PMN-MDSCs inhibit T cell immune responses, promote angiogenic factors, enhance the stem cell potential of tumor cells, and protect circulating tumor cells from host immune system attacks. This protective role aids in tumor metastasis and progression while conferring resistance to cancer immunotherapy (56–58). The C5a component of the complement system has emerged as a crucial immunoregulatory factor, capable of modulating various immune responses and influencing the activity of PMN-MDSCs (59). The findings from the study highlight a significant role for complement C5a in cancer progression, specifically in enhancing tumor growth and metastasis through the modulation of PMN-MDSCs. Recent research showed that by stimulating these immune cells, C5a aids the invasive capabilities of cancer cells via NETs, with the NETosis process being dependent on the high mobility group box 1 protein (HMGB1) produced by tumors. The study also identified that C5a promotes the expression of HMGB1 receptors, TLR4 and RAGE, on the surface of PMN-MDSCs, which could further facilitate tumor survival and immunity evasion. The experiments conducted in mouse models of lung metastasis showed that inhibiting C5a, its receptor, or the NETosis process resulted in reductions in circulating tumor cells and overall metastatic burden, suggesting potential therapeutic avenues. Moreover, clinical relevance was supported by findings that PMN-MDSCs from lung cancer patients exhibited increased migration and NETosis in response to C5a, with elevated levels of myeloperoxidase (MPO)-DNA complexes—a hallmark of NETosis—correlating with C5a levels in these patients. This research underscored the dual role of PMN-MDSCs in both immune suppression and facilitating tumor spread through NET formation, suggesting that targeting the C5a pathway could represent a promising strategy for developing innovative cancer therapies aimed at enhancing anti-tumor immunity and reducing metastasis (60).

#### 3.1.2 Recurrence and metastasis of tumors can be stimulated by NETs resulting from surgery and under stress

The release of NETs can stimulate tumor recurrence and metastasis by fostering an inflammatory environment that promotes the survival and proliferation of cancer cells (61). In a recent research, there remained the possibility of recurrence for limited-stage breast cancer after surgery, and the window of opportunity for recurrence was nearly always between six and twelve-months following surgery (62, 63). Researchers hypothesized that this phenomenon could create circumstances for the survival and spread of tumor cells by influencing patients' stress responses and wound healing processes during surgery (61). A worse prognosis and increased risk of tumor recurrence were associated with patients with colorectal liver metastases who experience NETs surge following surgical removal of all malignancies (64, 65). Additionally, in mice models, it has been demonstrated that NETs could stimulate peritoneal metastases (66, 67). Moreover, there had been a greater probability of abdominal metastasis or recurrence following surgery for patients with colorectal cancer who had an increase in low-density neutrophils (LNDs) during the perioperative phase that were able to spontaneously produce NETs. While a number of tumor cells were captured and shielded by the newly generated NETs, the LDNs in patients' perioperative stress states prevented killer T cells from killing (66).

#### 3.1.3 NETs aid in the survival of CTCs

As part of their medical treatment, the majority of individuals with cancer undergo surgery no fewer than once (68). Circulating tumor cells have the capability to enter the bloodstream and disseminate to distant sites, facilitated by surgical manipulation of the primary tumor. This phenomenon has been linked to potential advancements in tumor progression and the spread of metastases (69, 70). Researches have shown that surgical incisions can unintentionally promote the growth and spread of tumor (70). Postoperative infection represents a considerable concern, with a notable incidence rate, presenting a significant complication for cancer patients (71). This infection could result from a compromised immune system linked to the cancer itself, including complications such as intestinal obstruction or pneumonia. Alternatively, it could also be triggered by immunosuppression induced by standard cancer treatments such as chemotherapy and surgery (72). These circumstances markedly elevate the risk of postoperative infection. Research has indicated that up to 40% of cases suffered from postoperative infections (73). Unfavorable oncological outcomes have been associated with significant postoperative infections (74). Specifically, surgical procedures have been identified as triggering the release of NETs. These NETs possess the capacity to interact with circulating tumor cells, thereby potentially augmenting their capacity for adhesion and invasion into adjacent tissues (75, 76). Chronic stress has been linked to persistent inflammation and weakened immune responses, both of which are known to promote the progression and metastasis of tumors. The induction of NETs due to stress could promote the migration and infiltration of cancer cells, thereby enhancing their spread to distant organs in the body (61). Jonathan Cools-Lartigue et al. have identified that NETs in the microvasculature of lung cancer cells produce DNA nets. These nets consist of chromosome-specific DNA and protein complexes. When released, DNA nets from NETs can interact with cancer cells, potentially facilitating tumor migration and invasion to distant sites in the body. Emphasizing the production and role of NETs shows potential in mitigating the progression and metastasis of lung cancer. Continued investigation via research and clinical trials has the potential to broaden therapeutic avenues for lung cancer patients, affirming NETs as a feasible target for intervention. This research may pave the way for novel treatment modalities focused on addressing NETs (77).

### 3.2 NETs help to develop tumor-associated thrombus

Research has demonstrated that NETs generated by neutrophils have been demonstrated to enhance the development of thrombosis in animal models. These NETs, which are made of tissue proteases, cytokines, and DNA, have the potential to contribute to venous and arterial thrombosis (29). Mantovani and colleagues were the first to report on the role of NETs in human thrombus formation. They found NETs components in venous and arterial thrombi retrieved from patients, suggesting that NETs may play a critical role in the early stages of thrombus formation (78). When platelets and red blood cells coagulate, the DNA skeleton that NETs produce not only acts as a reaction support point for them, but it also binds to tissue factor, the initiator of the exogenous coagulation pathway that originates from NETs. This increases the coagulation reaction’s efficiency and promotes thrombosis (78). Studies revealed that compared to the general population, individuals with cancer have a greater likelihood of developing venous and arterial thrombosis. This increased risk is attributed to various factors, with one important factor being that cancer itself promotes the formation of NETs through multiple mechanisms, thereby facilitating thrombus formation. Tumor cells can directly promote the formation of NETs. Additionally, platelets in cancer patients play a key role in promoting NETs formation. Certain cytokines, such as granulocyte-macrophage colony-stimulating factor (GM-CSF) and IL-17, have also been found to increase NETs production, thus promoting thrombosis (79–81). Research has shown that co-culturing human pancreatic cell lines with neutrophils can increase the release of NETs by neutrophils, and can promote the occurrence of thrombus formation. This finding further supports the important role of NETs released outside neutrophils in promoting the process of thrombus formation (82). Another study observed that neutrophils isolated from patients with gastric cancer exhibited enhanced NETs formation ability compared to healthy controls (80). This suggests that gastric cancer could promote the process of neutrophils releasing NETs through certain mechanisms, potentially increasing the risk of thrombosis. NETs are also present in the blood clots of cancer patients. NETs released from neutrophils have been found in different types of cancer, and these NET components can play a role in both arterial and venous thrombosis. Increased formation of NETs may contribute to an increased risk of thrombosis (83). Additionally, studies have shown a positive correlation between circulating H3Cit (the ionized form of histone H3) and coagulation activation markers such as thrombin-antithrombin complexes. This positive correlation suggests that NETs released by neutrophils can promote activation of the coagulation system, thereby increasing the risk of thrombosis (83). The MPO-DNA complex is made up of DNA, the enzyme granule protein myeloperoxidase (MPO), and NETs, which are released by neutrophils. Studies have shown that patients with myeloproliferative neoplasms with thrombosis have higher levels of MPO-DNA complexes, which may reflect an increased formation of NETs in these patients (84). Research has shown that levels of circulating NETs (including components such as H3Cit) are increased in cancer patients, which may promote activation of the coagulation system, making these patients more prone to venous thromboembolism (85). Therefore, elevated plasma levels of H3Cit may serve as a potential biomarker for assessing the risk of venous thromboembolism in cancer patients. Denisa D Wagner and colleagues’ research showed that in tumor-bearing mice, neutrophils enhanced the formation of NETs *in vitro* compared to control mice similar to cancer patients. This finding suggests that tumors may promote the generation of NETs by affecting the function of neutrophils, potentially increasing the risk of thrombosis. The researchers further observed elevated levels of H3Cit and extracellular DNA in the plasma in mouse models carrying tumors, leading to the occurrence of DNA-rich pulmonary thrombi *in vivo (* 86). Another study showed that levels of NETs biomarkers in the plasma and levels of NETs in arterial and venous thrombi were increased in mice carrying 4T1 tumors. Comparing to control mice, mice with tumors formed larger thrombi. Interestingly, DNase I was effective in reducing arterial thrombus formation in both tumor-bearing and control mice, but only had a reducing effect on venous thrombus formation in tumor-bearing mice. This suggests that DNase I may have different regulatory effects on different types of thrombus formation (81). In a mouse model study of pancreatic cancer, it was found that IL-17 can promote the formation of NETs released by neutrophils (87). This suggests that IL-17 may promote the occurrence of this inflammatory reaction in pancreatic cancer patients, which may in turn affect the occurrence of thrombosis. Another study observed increased levels of circulating neutrophils and plasma NET biomarkers in mice carrying human pancreatic tumors. At the same time, increased level of H3Cit was found in venous thrombi (88). These results suggest that pancreatic cancer could increase the risk of thrombosis by promoting the formation of NETs released by neutrophils. Jak2V617F mice are a commonly used model for myeloproliferative neoplasms, which simulate patients with the Jak2V617F mutation. Studies have shown that compared to control mice, these Jak2V617F mice exhibit increased levels of NETs formation. This indicates that these mice have more NETs released into the surrounding environment. Furthermore, it has been observed that these Jak2V617F mice display spontaneous pulmonary thrombosis, which may be attributed to the increased risk of thrombosis due to elevated NETs. In this model, treatment strategies targeting NETs have shown potential efficacy. Specifically, treatment with DNase I, deficiency of PAD4, and the JAK inhibitor Ruxolitinib have been found to reduce the occurrence of venous thrombosis in these Jak2V617F mice. DNase I degrades the DNA portion of NETs, PAD4 deficiency affects the formation of NETs, and the JAK inhibitor Ruxolitinib can decrease the risk of thrombosis by inhibiting the JAK-STAT signaling pathway to reduce inflammation in the body (89). Therefore, treatment strategies targeting NETs, such as the use of DNase I, PAD4 deficiency, or JAK inhibitors, may have potential benefits in reducing thrombosis in myeloproliferative neoplasm models, providing new insights for future clinical therapy.

### 3.3 NETs promote tumor growth by reawakening latent tumor cells

The majority of cancer patients ultimately die from the metastasis of distant tissues, rather than the primary tumor. The spread of cancer cells is a key process in cancer progression. Dormant cancer cells can remain in a quiescent state for extended periods of time after disseminating to other tissues, sometimes even for decades. These dormant cancer cells are sometimes described as ‘seeds’ asleep, and they are clinically undetectable (90, 91). When these dormant cancer cells are reactivated and awaken, they can lead to the recurrence of metastatic cancer. This phenomenon has been observed in many solid tumors, such as breast cancer, lung cancer, and so on (92–94). Although the understanding of the mechanisms and factors influencing cancer cell dormancy is limited, researchers are actively working to unravel this mystery in order to develop more effective cancer treatment strategies. Dormant cancer cells refer to a state in which cancer cells stop proliferating and spreading under certain conditions, entering a dormant state (90). These dormant cancer cells can survive in the body for a long time, waiting to be reawakened and start growing and spreading again at the ‘right moment’, leading to cancer recurrence and metastasis. In the early stages of cancer, slow-cycling cancer cells may spread early in the body and form tiny seeds in secondary organs, where they stay in specific ‘niches’ or ‘seeding sites’ awaiting the proper stimuli to reactivate them (90–93). The presence and reawakening of these dormant cancer cells are important factors leading to cancer recurrence and metastasis (93, 95, 96). The exact mechanisms underlying the awakening, re-initiation of proliferation, and metastasis of slow-cycling cells that have been overlooked by the immune system are still largely unknown.

Neutrophils play a crucial role in tumor development, especially in the relationship between inflammation and tumor progression (97). Neutrophils are part of the immune system and are extensively involved in regulating inflammation and infection (53). Their activities in the tumor microenvironment can impact tumor growth, spread, and metastasis (98). Neutrophils can influence tumor immune editing by forming NETs, affecting the interaction between the immune system and cancer cells, thus promoting the growth, invasion, and metastasis of cancer cells, leading to tumor deterioration and recurrence (4). Studies have found that NETs can awaken dormant cancer cells, causing these cancer cells to resume growth and spread, and thus may be one of the reasons for tumor recurrence and metastasis (4, 53, 99, 100). Inflammation plays an important role in the development of cancer, especially in the transition between cancer cell dormancy and metastasis (101). Studies have shown that in breast cancer survivors, elevated C-reactive protein levels are associated with a lower disease-free survival rate (102), indicating the impact of inflammation on cancer recurrence and metastasis. The long-term recurrence of breast cancer post-surgery highlights the presence of dormant cells in the body that can re-emerge under conducive conditions to form tumors. Studies have shown that during inflammation, NETs can trigger dormant breast cancer cells to exit their quiescent state and start proliferating (103). Additionally, research conducted by Arelaki et al. has demonstrated that NETs can promote the growth of colorectal cancer cells (104).

Albrengues and others proposed the seed and soil hypothesis in their research, referring to the tendency of tumor cells (seed) to migrate to specific organs in the local microenvironment that are favorable (soil). Among the many components in the tumor microenvironment, neutrophils and their products play a crucial role in tumor cell development, immune evasion, and metastasis. This suggests that neutrophils play a key role in regulating inflammatory responses in the tumor microenvironment and promoting the progression and metastasis of tumors. This research found that during the process of lung inflammation induced by lipopolysaccharide (LPS) or tobacco smoke, NETs are necessary to awaken dormant cancer cells and cause mouse metastasis. These NETs can concentrate neutrophil elastase (NE) and MMP 9 on their substrate-bound adhesive proteins, allowing for continuous cleavage to generate sites that trigger cancer cell awakening. These findings suggest that NETs may play an important regulatory role between lung inflammation and cancer metastasis. In the *in vitro* experiments of this study, researchers found that specific types of basement membrane proteins such as laminins-111, -211, -411, and -511 are crucial extracellular matrix (ECM) proteins that lead to the induction of cancer cell awakening by NETs. These proteins may play a significant role in promoting cancer cell awakening and metastasis. This research finding provides important references and insights for further exploration of the mechanisms of cancer cell dormancy and awakening as well as the development of related therapeutic strategies. The study further confirmed the role of NET-remodeled laminins in the process of lung inflammation, activating integrin α3β1 activation sites, initiating the FAK/ERK/MLCK/YAP signaling pathway inside cancer cells, leading dormant cancer cells to reawaken. Based on this discovery, researchers developed inhibitory antibodies targeting NET-remodeled laminins, which could prevent the awakening of dormant cancer cells under conditions of lung inflammation. The findings of this study provide important references for the future development of therapeutic strategies targeting cancer cell dormancy and awakening mechanisms, and may offer new treatment options for preventing cancer recurrence (105).

### 3.4 NETs stimulate angiogenesis

Pathological angiogenesis is a common phenomenon in the process of inflammation and tumor growth. Both inflammation and tumors can trigger inflammatory reactions, leading to local tissue hypoxia and nutrient deficiency (106). The growth and metabolism of normal cells and tissues require an adequate supply of oxygen and nutrients, and tumor cells are no exception. Hypoxia, cellular metabolic demand, and nutrient deficiency stimulate angiogenesis. The blood supply needed to sustain tumor growth is achieved through a process called angiogenesis. Tumor cells produce signaling molecules, such as vascular endothelial growth factor (VEGF), to promote the growth and twisting of surrounding blood vessels, increasing their density and allowing them to obtain more oxygen and nutrients. This process is known as angiogenesis, and tumors ensure they have sufficient blood supply by promoting angiogenesis, allowing them to continue growing and spreading. Angiogenesis plays a crucial role in tumor growth and metastasis (12, 107, 108).

Angiogenesis is a complex and precise process that requires the coordinated interaction of multiple cells and molecules. Endothelial cells (BECs) play a crucial role in this process. This process is activated by angiogenic factors such as vascular endothelial growth factor (VEGF), angiopoietins (ANGPT), platelet-derived growth factor (PDGF), basic fibroblast growth factor (bFGF), epidermal growth factor (EGF), and CXCL 8/IL-8. Additionally, there are also anti-angiogenic factors that can inhibit angiogenesis. These pro-angiogenic factors and anti-angiogenic factors interact and regulate each other to maintain a dynamic balance in the process of angiogenesis. In pathological conditions such as tumors and inflammation, this balance may be disrupted, leading to abnormal angiogenesis (109–111). Under normal circumstances, the expression level of ANGPT 2 is low, but in areas of inflammation and in tumors, its expression increases significantly. Studies have shown that in cancer, the levels of ANGPT 2 rise, which may promote angiogenesis, tumor growth, and metastasis. Weak ANGPT 1-TIE 2 signaling could lead to inactivation of the AKT signaling pathway, thereby activating Foxo 1 and promoting the expression of ANGPT 2. In this scenario, ANGPT 2 promotes phosphorylation of TIE 2 to compensate for inadequate activation of TIE 2 induced by ANGPT 1, leading to increased endothelial cell chemotaxis and tube formation (112, 113). This suggests that in pathological conditions, ANGPT 2 may play a crucial role in regulating angiogenesis and endothelial cell function.

In addition to its traditional functions of killing bacteria and regulating inflammation, neutrophils also have the ability to produce and release a wide range of pro-angiogenic factors. Among them, VEGF-A is considered one of the most effective pro-angiogenic molecules, and it is present in human neutrophils and plays an important role. VEGF-A can be released through various signaling pathways, including in response to stimuli such as N-formyl-methionyl-leucyl-phenylalanine (fMLF), tumor necrosis factor-alpha (TNF-α), lipopolysaccharide (LPS), granulocyte colony-stimulating factor (G-CSF), and phorbol myristate acetate (PMA). These stimuli can activate human neutrophils, leading to the release of VEGF-A, and subsequently participate in the processes of inflammation and tumor angiogenesis (114–116). Aldabbous et al.’s research demonstrated the direct role of NETs in promoting angiogenesis both *in vitro* and *in vivo*. Their findings suggest that NETs could enhance endothelial cell proliferation and tube formation, thereby facilitating the process of blood vessel formation. Researchers applied multiple immunohistochemical staining to analyze the spatial distribution of NETs and microvessels in patient tissue samples. They established a subcutaneous tumor model in mice to observe the impact of NETs on tumor growth and used immunohistochemical staining to observe changes in tumor microvessel density (76). In the study, multiple immunohistochemical staining techniques were used to analyze the spatial distribution of NETs and microvessels in patient tissue samples. Establishing a subcutaneous tumor model in mice can be used to observe the effect of NETs on tumor growth, and immunohistochemical staining can be used to observe and analyze changes in microvessel density in tumor tissue. By labeling proteins related to blood vessels or labeling vascular endothelial cells to quantify the number and density of vessels, researchers found that NETs promote neovascularization and play a role in the progression of gastric cancer. Furthermore, the study found that blocking NETs associated with reduced microvessel density significantly inhibited tumor growth in a subcutaneous tumor model in mice. Tumor volume and mass in the inhibited group decreased by 61.3% and 77.9%, respectively, compared to the control group. Inhibiting the activity of NETs can effectively suppress tumor growth and development. These results suggest a potential role for NETs in tumor growth and angiogenesis, providing new insights for the development of novel cancer therapeutic strategies targeting NETs (117). Matrix metalloproteinases (MMPs) play an important role in the process of angiogenesis. MMP-9 has a promoting effect in angiogenesis. MMP-9 can promote the release of vascular endothelial growth factor (VEGF) from the extracellular matrix, increasing its activity and accelerating the process of angiogenesis. It also regulates the interaction between VEGF and its receptors, further regulating the progression of angiogenesis. The addition of neutrophils to pancreatic cancer cells can increase the budding rate by more than 2.5 times, indicating that MMP-9 may promote endothelial cell migration. After treatment with bevacizumab (a VEGF inhibitor) and doxycycline (a drug that can effectively inhibit angiogenesis like an MMP-9 inhibitor) for 14 days, the tumor volume in pancreatic cancer mice significantly decreased. Additionally, the average blood vessel density in pancreatic cancer mice also significantly decreased (118). Therefore, targeting the tumor vasculature system may be an effective strategy for treating tumors, especially those that are insensitive to traditional chemotherapy and radiation therapy. Studying the characteristics and mechanisms of the tumor vasculature system can provide crucial insights for developing more effective drugs targeting the tumor vasculature system. Further research on the role of MMPs, NETs, and their interactions in modulating angiogenesis and tumor growth could provide valuable insights for developing novel therapeutic strategies targeting the tumor vasculature system.

NETs release is mediated through the activation of Tie 2. Angiopoietin Tie2 receptor is an important member of the angiopoietin family and plays a crucial role in regulating angiogenesis and inflammatory responses. The Angiopoietin Tie2 receptor is a dual-function receptor that can bind to two different ligands: angiopoietin-1 (Ang1) and angiopoietin-2 (Ang2). By binding primarily to its ligand Ang1, the Angiopoietin Tie2 receptor promotes vascular stability and repair, suppresses endothelial cell inflammatory responses, and regulates vascular wall permeability to maintain normal vascular function. In contrast, angiopoietin-2 regulates angiogenesis and inflammatory responses by interfering with Ang1/Tie2 signaling. The role of Ang2 is complex, as it may promote angiogenesis and inflammatory responses in some situations while inhibiting angiogenesis and increasing vascular permeability in others. The expression of Tie2 receptor in neutrophils may also influence the function and activity of neutrophils. Induction of angiopoietin Ang1 and Ang2 may trigger neutrophil production and release of platelet activating factor, regulate the expression of the β2 integrin complex (CD11/CD18), increase neutrophil chemotaxis, and other inflammatory activities. These actions may contribute to the regulation and response of neutrophils in inflammatory and immune reactions (119).

The irregular structure and leaky nature of tumor vasculature make it a vulnerable target for treatment. It is particularly suitable for destruction mediated by NETs. Studies have shown that NETs may promote tumor invasion and metastasis by affecting endothelial cell-cell contacts and increasing permeability. NETs can affect endothelial cell-cell contacts and increase permeability, including in the context of metastasis. Researchers found complement system activation in melanoma patients and mouse melanoma samples, highlighting tumor endothelium as the starting point of complement cascade. Complement-derived C5a promotes neutrophil recruitment, leading to neutrophil activation and release of NETs. Positioned near the vessel wall, NETs open the endothelial barrier, enhancing vascular leakage, promoting melanoma cell invasion and systemic dissemination. Further investigation revealed that neutrophil depletion in animals lacking C6 or deficiency in membrane attack complexes (MAC) formation can protect the vascular endothelium, preventing melanoma cell intravasation. Thus, inhibiting MAC-mediated neutrophil activation may be an effective strategy to eliminate melanoma hematogenous dissemination (120). Therefore, targeting tumor vascular disruption mediated by NETs may be an effective therapeutic strategy. By blocking the formation or activity of NETs, their impact on the tumor vascular system can be reduced, thereby decreasing the growth and spread of tumors. This therapeutic approach may provide a new avenue for treatment, especially for tumor types that are resistant to conventional treatment methods. Thus, research and development of therapies targeting NETs-mediated tumor vascular disruption may have significant clinical significance in the field of cancer treatment.

### 3.5 NETs in Regulating T cells in tumor microenvironment

Neutrophils, essential to the immune system, act as the initial responders to infections, especially bacterial ones (24, 25, 28). Researchers have demonstrated various interactions between neutrophils and T lymphocytes, along with their associated products. Notably, NETs can impact T lymphocytes through mechanisms such as direct contact, cytokine release, and modifications to the local microenvironment. Within the tumor microenvironment, these interactions can profoundly influence tumor progression and the effectiveness of immunotherapy. Consequently, it is essential to gain a comprehensive understanding of how NETs regulate T cells and how T cells influence NET formation to develop more effective immunotherapeutic approaches (14, 25, 48).

The findings from the study by Kati Tillack et al. have provided a compelling insight into the complex roles that neutrophils play beyond their traditional functions in pathogen elimination. By identifying the interaction between NETs and T cells, the research underscored a novel mechanism through which the innate and adaptive arms of the immune system communicate and cooperate. One key finding from the study was that NETs can reduce the activation threshold for T cells, allowing these adaptive immune cells to respond more effectively even in the presence of suboptimal stimuli. This suggested that NETs can enhance immune protection during infections by making T cells more responsive. The research emphasized the importance of the physical interaction between NETs and T cells for this priming effect, with signaling through the T cell receptor (TCR) being crucial for a robust immune response. Interestingly, the study also discovered that Toll-like receptor 9 (TLR9) does not contribute to this priming process, indicating that NETs influence T cell activation through other, as yet unidentified, signaling pathways. This opens new research avenues to explore alternative mechanisms by which NETs affect T cell function. The study enriches our understanding of the interplay between innate and adaptive immunity, highlighting the multifaceted role of neutrophils. It underscores the need for further investigation into the mechanisms through which NETs affect immune responses and opens up promising avenues for developing novel therapeutic interventions (121).

In a recent study, researchers proposed that NETs interact directly with infiltrating T cells, contributing to the establishment of an immunosuppressive tumor microenvironment. To facilitate the development of a NET-rich tumor microenvironment, researchers conducted liver ischemia/reperfusion (I/R) experiments in a cancer metastasis model and also injected NETs directly into subcutaneous tumors. The findings indicated that within this NET-rich tumor microenvironment, the majority of CD4^+^ and CD8^+^ tumor-infiltrating lymphocytes expressed a variety of inhibitory receptors and exhibited signs of functional and metabolic exhaustion. The application of DNase to target NETs in mice resulted in decreased tumor growth, reduced NET formation, and increased levels of functional T cells. *In vitro* experiments demonstrated that NETs contain the immunosuppressive ligand PD-L1, which contributes to T cell exhaustion and functional impairment. The use of PD-L1 knockout NETs or co-culturing NETs with PD-1 knockout T cells effectively negated this effect. Furthermore, elevated levels of soluble PD-L1 and myeloperoxidase-DNA (NETs markers) were observed in the serum of patients who had undergone resection surgery for colorectal liver metastasis. Neutrophils isolated from these patients’ post-surgery were found to form NETs, leading to exhaustion and functional impairment of CD4^+^ and CD8^+^ T cells. Following this, the researchers administered a PD-L1-blocking antibody during liver I/R. After a single dose of anti-PD-L1 was given during surgery, tumor shrinkage was noted three weeks later, accompanied by an increase in functional T cells within the tumor microenvironment. These results indicated that NETs can suppress T cell responses through metabolic and functional exhaustion, thereby facilitating tumor growth. Additionally, the deployment of DNase or targeting PD-L1 in NETs during surgery both contributed to reduced tumor growth, presenting a promising approach for enhancing immune competence in the tumor microenvironment (122).

## 4 NETs in gynecological cancers

### 4.1 NETs in cervical cancer

With almost 270,000 fatalities annually, cervical cancer ranks as the third most frequent malignancy worldwide. Even with the great improvements in surgery, chemoradiotherapy, and immunotherapy, approximately 40% of patients will eventually die from a recurrence following treatment with the intention of curing their illness (123, 124). Based on the anatomical extent of the tumor, the Union for International Cancer Control (UICC), tumor, nodes, metastasis (TNM) and the Federation International of Gynecology and Obstetrics (FIGO) classification system are used for prognostication and treatment recommendations in cervical cancer (125, 126). However, the clinical outcome varies significantly among patients with the same tumor stage (123, 126). In recent years, there has been a shift in the understanding of cancer development and progression from a focus solely on the tumor itself to a more holistic view that includes the entire microenvironment in which the tumor exists. The tumor microenvironment encompasses various factors such as immune cells, blood vessels, and extracellular matrix components, all of which play critical roles in supporting tumor growth and invasion (98, 127). It is now known that the biological behavior of invasion, metastasis, and tumorigenesis is caused by a disturbance of the dynamic equilibrium between the immunological components of the host and the tumor cells. Unbalance and ongoing interplay between malignant tumor cells and distinct stroma and immune cell subsets of the surrounding immunological microenvironment result in tumor progression (128). In contrast to the current histopathological techniques used for staging different cancers, such as lung cancer (129) and breast cancer (130), plenty immune-mediated statistics studies have found that the type, density, and location of immune cells in the tumor microenvironment serve as superior predictors of patient survival (98, 131, 132).

Researchers detected the formation of NETs in the tumor nests and stroma of 126 patients with cervical cancer using multiplex quantitative immunofluorescence technology, and discovered that: higher densities of stromal PMNs and NETs were correlated with poor outcome in a retrospective cohort of patients with cervical cancer, and high stromal NETs density was an independent prognostic factor for RFS. A closer examination of its relationship to the clinicopathological features of individuals with cervical cancer revealed that: the elevated density of stromal CD66b^+^ cells was substantially correlated with the clinical stage (*p*=0.006). No correlation was observed between lymph node involvement, histology, or pathologic grade and stromal CD66b^+^ cells. The results of the study indicate a positive correlation between the advancement of cervical cancer and a high density of neutrophils in the stromal tumor tissue. No statistically significant distinction was found between patients at different stages who had high or low concentrations of stromal CD66b^+^ cells. The following conclusions were drawn from the investigation of the relationship between patient survival without recurrence and the concentration of NETs in tumor communities and tumor stroma. To further elucidate the effect of neutrophils affecting patient outcome, the researchers expanded this observation to include cervical cancer in Staging I–IV. The researchers found that the stromal tissue contained the majority of neutrophils. It was noted that neutrophils were primarily found in the stromal tissue. The association between short RFS and an elevated stromal CD66b^+^ neutrophil density was shown to be significant, as opposed to a high tumor nest CD66b^+^ neutrophil density. A favorable RFS was connected with low stromal CD66b^+^ neutrophil density in univariate analysis, but in multivariate analysis, this component did not function as an independent predictor (133). Further researches are necessary to determine the significance of NETs activity associated with tumor boundary movement, as suggested by this finding, as demonstrated in **Table 1**. Using TNM staging rather of FIGO staging resulted in a deficiency of certain clinical prognostic variables. A further limitation of the study was the small number of participants, which could account for the lack of statistically significant differences in the clinical phase of subset evaluation of high- and low-density NETs. Further researches are necessary to elucidate the underlying process.

**Table 1:** Neutrophil extracellular traps (NETs) in gynecological cancer and areas for further research.

Cancer Type	Technology Used	Clinicopathological Features	Key Findings	Further Research Needs	References
**Cervical Cancer**	Multiplex quantitative immunofluorescence	Elevated density of stromal CD66b^+^ cells correlated with clinical stage;	Higher densities of stromal PMNs and NETs correlated with poor outcomes;	Investigate the activity of NETs in relation to the movement of tumor boundaries;	(133)
		No correlation found between stromal CD66b^+^ cells and lymph node involvement, histology, or pathologic grade.	High stromal NETs density was an independent prognostic factor for recurrence-free survival.	Address limitations of TNM staging vs. FIGO staging;	
				Larger sample sizes needed to discern clinical prognostic differences;	
				Further research required to elucidate underlying processes.	
**Endometrial Cancer**	Fluorescence microscopy	Stage IA primary endometrial cancer; Peripheral blood analysis of 123 patients.	Neutrophils in patients showed altered morphology and loss of granules; Increased MMP-1 levels due to heightened reactive oxygen species production; Decreased IL-2 levels, reducing cytotoxic activity induction; Marked rise in G-CSF levels, affecting neutrophil function.	Clarification of how altered neutrophil function impacts disease progression; Longitudinal studies to assess changes in neutrophil behavior over time; Exploration of therapeutic implications based on neutrophil alterations.	(134)
	Antibodies targeted against citH3; IHC(Immunohistochemistry); IF (Immunofluorescence) analyses; Serum cfDNA and cfmtDNA analysis.	Endometrial cancer tissue samples; Healthy endometrial tissue as controls.	Presence of NETosis biomarker citH3 in most tumor tissue samples; Positive link between citH3 and cfDNA concentration in EC serum; Negative correlation between cfmtDNA and citH3 in healthy serum.	Determine prognostic significance in different tumor types; Mechanisms of NETosis in EC; Clinical Utility of NETosis Biomarkers; Impact of Treatment on NETosis.	(135)
**Ovarian Cancer**	Examination of ascites for NE levels, mitochondrial DNA, and their impact on NETosis.	Study focused on ovarian cancer ascites, investigating associations with progression-free survival and chemotherapy-refractory illness.	High NE levels correlated with shorter median PFS and higher risk of progression;	Explore mechanisms of neutrophil cross-activation in ovarian cancer ascites;	(142)
			mtDNA in ascites linked to poor prognosis and chemotherapy resistance;	Investigate therapeutic strategies targeting NETosis pathways;	
			In vitro evidence of ascites stimulating NETosis and neutrophil activation.	Validate mtDNA and NE levels as prognostic markers in larger cohorts;	
				Understand impact of ascites on tumor microenvironment and metastasis.	
	Examination of NETs in omental tissue;	Early-stage ovarian cancer patients; nonmetastatic ovarian cancer;	NETs within the omentum as a response to early-stage ovarian tumors;	Mechanisms of neutrophil recruitment; Long-term effects of NETs inhibition;	(67)
	Animal models (mice) and human patient samples used for research.	Neutrophil recruitment to omentum; omental metastases.	NETs presence in omental tissue of both humans and animal models;	Clinical trials to validate NETs inhibitors' efficacy in humans.	
			NETs inhibitors reduce omental metastases in animal models.		
	Quantification of H3Cit-DNA, dsDNA, and CA 125 plasma levels in peripheral blood samples	199 patients undergoing primary surgery for adnexal tumors	Patients with borderline or malignant ovarian tumors did not show higher levels of H3Cit-DNA or dsDNA plasma compared to benign tumor patients.	Investigate other potential biomarkers or combinations of biomarkers for more accurate diagnosis and prognosis of ovarian tumors.	(144)
			Borderline and ovarian cancer groups exhibited higher CA-125 levels.	Explore the mechanisms behind CA-125 elevation in different stages and subtypes of ovarian tumors.	
			CA-125 levels did not significantly affect the survival analysis of malignant ovarian tumors.	Conduct longitudinal studies to validate these findings and assess their clinical utility in guiding treatment decisions and monitoring disease progression.	

### 4.2 NETs in endometrial cancer

As for endometrial cancer, as demonstrated in **Table 1**, fluorescence microscopy was used by TV Abakumova et al. to investigate neutrophils and their capacity to create extracellular traps in the peripheral blood of 123 patients diagnosed with stage IA primary endometrial cancer. Enhancement of both aerobic and anaerobic cytotoxicity and phagocytosis, as well as a decrease in net activity, have all been demonstrated to occur as the relative quantity of neutrophils increases. Observations of altered neutrophil secretory activity included elevated MMP-1 levels, most likely from higher reactive oxygen species generation, lowered IL-2 levels, which is an inducer of cytotoxic action, and a marked rise in G-CSF levels. Patients with stage IA carcinoma of the endometrium have neutrophils that have changed in morphology and lost granules. They detected alterations in neutrophil secretory activity, marked by elevated MMP-1 levels—likely resulting from heightened the amount of reactive oxygen species production—decreased IL-2 levels, a trigger of cytotoxic activity, and a marked rise in G-CSF levels. Granule loss and a change in form are features of the neutrophil structure among individuals with stage IA endometrial cancer (134).

Using antibodies targeted against citH3, the team of investigators carried out IHC and IF analyses of EC tissue and endometrial tissue from healthy individuals as controls for the purpose of assessing the presence of NETosis signatures in EC. They identified that leukocytes infiltrates that were positive for the NETosis biomarker identification citH3 were present in most tumor tissue samples. The general trend from grade G1 to grade G3 was upward. Nevertheless, there was no correlation found between the serum levels of citH3 and its tissue staining. This might be because the processes by which cfDNA is cleared in tissue differ from those in serum, or it could be because blood and tissue samples are collected at separate times. It has been discovered that endometrial cancer patients can be distinguished from individuals who are healthy using serum levels of cfDNA and cfmtDNA. In endometrial cancer samples, serum citH3 levels were significantly higher. In EC serum, there was a positive link between citH3 and cfDNA concentration, while in healthy individuals’ serum, there was a negative correlation between cfmtDNA and citH3. Through the use of serum cfDNA and cfmtDNA in combination with serum citH3 concentration, these novel non-invasive indicators of NETosis in endometrial cancer can now be used for prognostic and diagnostic purposes as well. Further research is required to determine the prognostic significance of the combined indicators in different tumor types (135).

### 4.3 NETs in ovarian cancer

**Figure 3:**
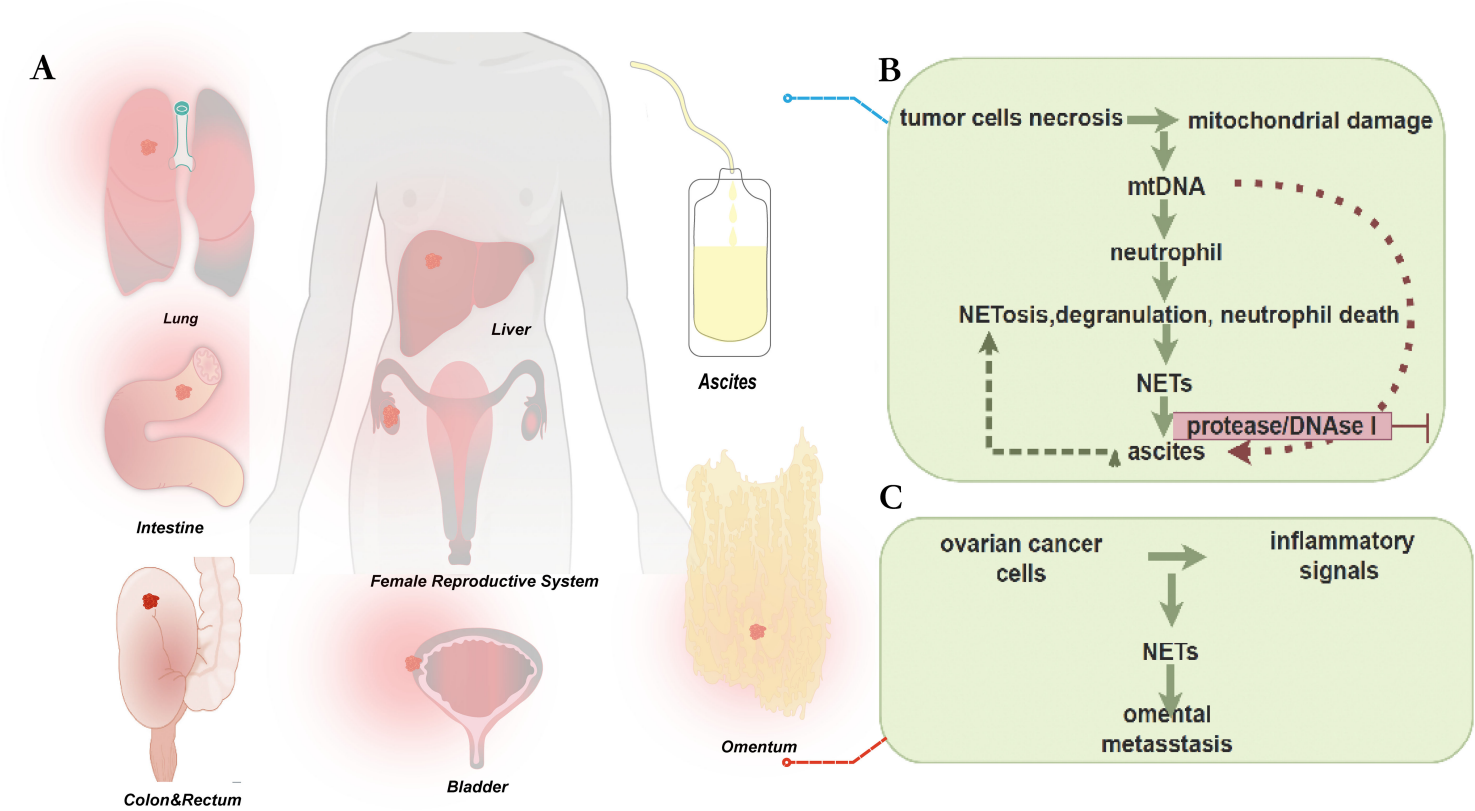
**(A)** Common routes of metastasis in epithelial ovarian cancer include several ways described as follows. Direct invasion of adjacent organs is a common route, especially in advanced stages of the disease. In addition, implantation metastasis in the peritoneum and within the abdominal cavity is also a common route, which can lead to the formation of peritoneal carcinomatosis. Lymphatic spread often occurs in the pelvic and abdominal lymph node regions. Hematogenous spread may also carry cancer cells to other organs, such as the liver, lungs, or other organs. Understanding these routes of metastasis is important for the diagnosis and treatment of epithelial ovarian cancer. In cancer research, NETs have attracted a lot of attention, while research on ovarian cancer is still in its early stages. **(B)** Detect levels of NE (a marker of neutrophil extracellular trap (NET) release, occurring in processes such as NETosis, degranulation, and neutrophil death) and mtDNA (a molecular pattern associated with mitochondrial damage, released by tumor cells during necrosis) in ascites from ovarian cancer patients. Levels of mtDNA and NE are significantly positively correlated with shorter PFS. Ascites can attract neutrophils and induce NETosis, potentially stimulating neutrophil responses and promoting metastasis. Ascitic mtDNA levels are associated with poor prognosis, potentially related to platelet-derived mtDNA and microparticles. There are multiple platelet activation pathways in the tumor microenvironment, which may serve as prognostic markers and/or therapeutic targets. **(C)** The peritoneal spread of ovarian cancer is found to be a deliberate process, in which cancer cells cooperate with host cells to create favorable conditions in predetermined locations. Research has shown that neutrophils respond to early ovarian tumors by producing NETs in the omentum, potentially manufacturing NETs in the omentum before metastasis. Ovarian cancer cells adhere to NET-positive neutrophils and omental metastasis, but tumor growth is significantly reduced in mice lacking NET formation. These findings suggest that intervening in NET formation may prevent omental metastasis and could help in understanding ovarian cancer treatment and related complications.

Of all the gynecological malignant tumors, epithelial ovarian cancer, especially HGSOC, has the greatest fatality rate (136, 137). While platinum-based the first-line chemotherapy medicines have demonstrated notable anti-tumor efficacy in numerous solid tumors, the absence of precise and accurate diagnostic tools and recurrence biomarkers has hindered the improvement of the five-year survival rate for ovarian cancer over the last ten years (138). Due to the fact that most patients get diagnosed at advanced stages, when they have widespread metastases throughout the peritoneal cavity, this is one of its most significant disadvantages (136–138). Accordingly, most OC tumor spread follows peritoneal fluid dynamics, while it can also propagate via systemic or lymphatic channels. The significance of deepening our comprehension of the constituent parts and mechanisms of peritoneal fluid is underscored by its distinctive manner of metastasis, which renders it the most representative biological fluid in the OC tumor milieu (139, 140). Furthermore, recurrence and resistance to chemotherapy are the primary reasons of death from ovarian cancer (141). A pressing issue that needs to be resolved is the research on the molecular mechanisms of metastasis, recurrence, and treatment resistance. In cancer research, NETs have garnered a lot of interest, while OC research is still in its early stages. Current research on the connection between NETs and OC was included in this review (**Figure 3**).

In an attempt to gain further insight into the possible significance of NETs markers in OC diagnosis and/or prognosis, recently published studies has inquired into whether they can serve as prognostic markers. The study by Singel et al. examined the levels of NE (markers of released neutrophil granule products occurring in NETosis, degranulation, and neutrophil death) and mtDNA (molecular patterns associated with mitochondrial damage released by tumor cells during necrosis) in ovarian cancer ascites. There was a non-linear association between ascites NE levels and PFS, with patients with the highest levels having a significantly shorter median PFS and a greater risk of disease progression within 12 months following initial surgery. It’s noteworthy to note that mtDNA has been identified as a NETosis activation stimulant. When the trial was limited to a period of 12 months following surgery, which is clinically characterized as chemotherapy-refractory illness, survival analysis showed a significant positive relationship between mtDNA and NE levels and shorter progression-free survival. Moreover, it was shown *in vitro* that ascites can draw neutrophils and cause NETosis, indicating that mtDNA and other elements found in this bodily fluid may stimulate neutrophil reactions and encourage metastases (142). Thus, as demonstrated in **Table 1**, it was suggested that these pathways could function as possible indicators of prognosis and/or targets for treatment. Subsequent investigation revealed that a poor prognosis was linked to the ascites mtDNA level (but not the gDNA level because platelets are enucleated). This indicates that the mechanism of neutrophil cross-activation and NETosis in EOC ascites may be related to platelet-derived mtDNA and microparticles. Furthermore, the investigators noticed significant alterations in the capacity of enzyme inhibitors such as protease and DNAse I to stop platelet activation in ascites supernatants, indicating the presence of several mechanisms for platelet activation in the tumor microenvironment. As a result, they postulated that these pathways could serve as prognostic markers and/or therapeutic target.

In contrast to the earlier research conducted by Muqaku et al. (143) employed a comparable methodology to investigate the function of neutrophils in either stimulating or impeding tumor cell activity inside the tumor-associated microenvironment of high-grade serous ovarian cancer. NET-related molecules (NE and MPO) and local inflammatory markers (calprotectin heterodimers, including S100A8 and S100A9, which are also thought to be cytoplasmic markers of NETs) were found to be more abundant in ascites samples from patients with non-miliary metastases than in ascites samples from patients with miliary metastases. Several apparent disparities can be explained by the models suggested by the current data. Three layers make up the model: (1) hypoxic cellular stress initiates the development of NETs; (2) NETosis establishes distinctive microscopic features linked to certain biomarker profiles; and (3) NETosis modulates the adaptive immune system to enhance overall survival (142, 143).

Extraperitoneal spreading of ovarian cancer is characterized by a significant affinity for the omentum and has frequently been explained as a passive process that is driven by the mechanical movement of peritoneal fluid. Nevertheless, recent research on different kinds of tumors has demonstrated that metastatic tropism is a deliberate process whereby the tumor collaborates with different host cells to create favorable conditions at the intended site before colonization. Researchers have found that NETs indicators are present in the omentum of mice with ovarian tumors and people with early-stage ovarian cancer, and that these markers appear to be colonized at the site. However, in healthy women and animals without tumors, these markers are generally absent.

It is shown that the creation of NETs in the omentum prior to metastasis is an early response of neutrophils to ovarian tumors in the abdominal cavity by Lee, W., et al’s findings, which imply that early ovarian tumors could release inflammatory signals to recruit neutrophils to the omentum and trigger NETs secretion (67). Interestingly, they found NETs in the omentum of patients with nonmetastatic early-stage ovarian cancer and in animals with ovarian cancers. Notably, NETs were found in the omentum of both patients with nonmetastatic early OC and mice with ovarian malignancies. Based on these data, it is possible that neutrophils responding early to intra-abdominal ovarian malignancies create NETs in the omental niche prior to metastases. Notably, ovarian cancer cells attached to NET-positive neutrophils and omental metastases but not primary tumor growth were markedly reduced in neutrophil-specific Padi 4^−/−^ mice defective in NETs formation but with normal white blood cell numbers and neutrophil chemotaxis. Omental metastases were dramatically decreased by NETs inhibitor therapy. When considered collectively, these findings raise the possibility that neutrophil inflow into the omentum may be a need for the development of premetastatic OC foci and imply that omental metastasis is prevented by interfering with NETs formation (67). Insights into ovarian cancer treatment and associated comorbidities may be gained from additional research into this remarkable host defense system.

Neutrophils with a variety of protumorigenic and antineoplastic characteristics are activated by stimuli within the tumor microenvironment. Through the quantification of H3Cit-DNA, dsDNA, and CA 125 plasma levels in peripheral blood samples taken from 199 patients undergoing primary surgery for adnexal tumors, researchers discovered that patients with borderline or malignant ovarian tumors did not have higher levels of H3Cit-DNA or dsDNA plasma than patients with benign tumors. The borderline and ovarian cancer groups had higher CA-125 levels, however the researchers concluded that this did not affect the survival analysis of malignant ovarian tumors (144).

## 5 Future prospects: the emergence of new technologies and devices in tumor treatment

The preceding discourse highlighted the potential of cancer surgery to inadvertently trigger the release of circulating tumor cells, thereby fostering tumor expansion and metastasis. Postoperative infections, a frequent complication, hold significant implications for oncological prognosis, particularly when infections escalate in severity. Neutrophils have been identified as key facilitators of cancer progression through the elaboration of NETs, which have been shown to interact with circulating tumor cells resulting in enhanced tumor cell adhesion and invasiveness. Consequently, these interactions have been linked to a less favorable prognosis for affected individuals. In light of recent advances in surgical treatment concepts and ongoing technological progress, a multitude of minimally invasive treatment modalities have surfaced. Presently, a host of ailments can be addressed through minimally invasive or non-invasive surgical interventions. These cutting-edge techniques employ thermal or cryogenic energy to induce necrosis within specified tissues, encompassing methodologies like focused ultrasound ablation, microwave ablation, radiofrequency ablation, and cryoablation (145–147).

### 5.1 High-intensity focused ultrasound

High-Intensity Focused Ultrasound (HIFU) is an advanced non-invasive thermal ablation modality that harnesses the power of high-energy ultrasound waves to precisely alter the architecture of targeted tissues, thereby enabling localized ablation therapy. By delivering focused ultrasound energy externally, HIFU triggers thermal necrosis within the target area. Moreover, when integrated with Magnetic Resonance Imaging (MR-HIFU), this technology allows for precise measurement and monitoring of temperature variations within the target tissue and its adjacent structures. This real-time feedback enables clinicians to make necessary adjustments during the treatment procedure (148, 149). Currently, FDA-approved HIFU applications encompass a wide range of clinical indications, including treatment of uterine fibroids, neurological disorders, as well as tumors affecting the prostate, breast, liver, and pancreas, among other anatomical sites (150). A comprehensive analysis was conducted on 153 cases of residual/recurrent cervical cancer within the previously irradiated region who received HIFU treatment. The investigation unveiled that following HIFU treatment, the objective response rate was 23.5% with a disease control rate of 93.5%. Notably, the median progression-free survival (mPFS) and median overall survival (mOS) were recorded at 17.0 months and 24.5 months, respectively. Moreover, it was observed that patients with lesions ≥1.40 cm prior to HIFU treatment and a post-treatment shrinkage rate ≥30% exhibited improved mPFS and mOS. Similarly, patients with post-treatment lesions ≤1.00 cm displayed increased mPFS, suggesting that HIFU possesses the ability to effectively enhance local control rates and extend patient survival (151). The findings indicate that HIFU treatment could emerge as the favored choice for individuals grappling with residual disease or recurrence post-radiotherapy for cervical cancer. Furthermore, all treatment-related adverse events were confined to mild complications such as skin burns, abdominal pain, and discharge. A recent investigation analyzed the outcomes of eight patients with recurrent ovarian cancer or metastatic pelvic tumors who underwent treatment. The study revealed that the pain relief rate among patients reached 60%, and there was an observable improvement in their short-term quality of life. The primary side effect of HIFU treatment was localized pain, with all patients reporting pain scores below 4 following the procedure. Additionally, all pain subsided within the first day after HIFU treatment. Notably, the study did not identify any instances of severe complications such as skin burns, intestinal perforation, or nerve damage. These findings suggest that HIFU may present a promising therapeutic approach for recurrent ovarian cancer, as well as metastatic pelvic tumors stemming from cervical cancer, endometrial cancer, and rectal cancer (152).

It is imperative to conduct additional research on the impact of high-intensity focused ultrasound on neutrophils and NETs. The necessity for further investigation lies in validating the potential effects of high-intensity focused ultrasound treatment on neutrophil function and the release of NETs, as well as understanding how these impacts may influence tumor growth and patient outcomes. By delving deeper into the relationship between high-intensity focused ultrasound treatment and the immune response, a comprehensive understanding of the therapy’s effects on the tumor microenvironment can be achieved, offering valuable insights to guide clinical practice and refine cancer treatment strategies. Future research endeavors should consider the intricate mechanisms of interaction between high-intensity focused ultrasound treatment and the immune system to effectively address challenges in cancer treatment.

### 5.2 Histotripsy

In the realm of clinical practice, the conventional method of focused ultrasound ablation primarily entails the induction of coagulative necrosis in tumor cells within the specific target region through thermal mechanisms. Nevertheless, the efficacy of this approach may be impacted by the vascular supply to the tumor, thus increasing the risk of incomplete necrosis. Additionally, prolonged exposure to thermal energy poses a potential threat of harm to surrounding healthy tissues. In order to achieve successful tumor ablation without compromising normal tissue integrity, investigators are delving into the possibilities of leveraging the non-thermal effects of focused ultrasound in disease treatment.

Histotripsy is an innovative non-invasive technology that utilizes focused ultrasound, similar to HIFU, yet with a fundamentally distinct mechanism which primarily involves mechanical effects at the cellular level for tissue destruction. This cutting-edge approach is rooted in the principles of non-thermal injury and non-ionizing radiation, employing a non-invasive, focused ultrasound methodology. By utilizing a low duty cycle (<1%) and short duration (microseconds to milliseconds) pulse ultrasound, this technique minimizes heat deposition while harnessing high peak negative pressure (>10 MPa) to induce cavitation bubbles within the target area, effectively pulverizing tissue into subcellular structures (153, 154). Furthermore, real-time ultrasound imaging serves as a valuable tool for guiding and monitoring the tissue destruction process. Histotripsy, in contrast to various other minimally invasive techniques, offers the distinct advantage of being non-invasive. This advanced technology is distinguished by its ability to precisely target and fragment solid tissues, such as tumors, effectively transforming them into acellular homogenate. The resulting fragments are subsequently absorbed by the body within a relatively short period of 1-2 months, leaving behind minimal scars (153, 155, 156).

To date, Phase III human clinical trials have been carried out to evaluate the safety and viability of Histotripsy in patients. Notably, a trial conducted in Barcelona (NCT03741088) involved the treatment of 11 liver tumor patients using the Vortx Rx device, with no reported adverse events. Following a two-month observation period, the average tumor shrinkage rate was found to be 71.8%, suggesting promising initial safety and effectiveness of the treatment in hepatic histotripsy (157). Since 2021, the United States and Europe have commenced two Phase I clinical trials exploring the efficacy of Histotripsy technology in the ablation treatment of primary and metastatic liver lesions. The outcomes of these trials have shown great promise, capturing the interest of the Food and Drug Administration in the United States. As a result, the FDA has fast-tracked the approval process for this cutting-edge technology. Histotripsy has demonstrated the ability to elicit strong innate and adaptive immune responses in animal models of melanoma and liver cancer. Evidence indicates that histotripsy leads to a significantly higher level of immune cell infiltration compared to radiation therapy or radiofrequency ablation. Additionally, histotripsy has been shown to provoke robust systemic anti-tumor immune responses and abscopal effects. Notably, histological examination of flank tumors has revealed a marked reduction in lung metastases compared to control groups. While the exact mechanisms are not yet fully understood, the overall therapeutic effects of histotripsy extend beyond local tumor ablation and may provide additional benefits while improving clinical outcomes for cancer patients. Further research is necessary to comprehensively elucidate its impact on formation of new tumor nodules. Despite substantial progress in the technical, preclinical, and clinical realms of histotripsy, significant future work remains in technology development, preclinical studies, and human research before histotripsy can become a widely adopted clinical treatment modality (158).

Future studies should place a strong emphasis on examining the effects of HIFU and histotripsy on the immune response in the tumor microenvironment, with a specific focus on neutrophil activity and the release of NETs. A deep understanding of the interactions between these therapies and the immune system is essential for improving treatment approaches, maximizing efficacy, and reducing risks. Progress in this area depends on conducting thorough research to uncover the mechanisms by which these treatments impact the tumor microenvironment and immune responses. This will establish a robust foundation for future developments in personalized medicine and immunotherapy.

### 5.3 NETs as potential cancer therapy targets

In the realm of cancer therapy, the exploration of NETs as potential targets presents an intriguing avenue for enhancing the immune response against tumors and potentially improving the effectiveness of current treatment modalities. This focus on disrupting NETs formation or function within the context of cancer aims to shift the delicate immune balance towards a more robust anti-cancer response. Researchers are delving into the intricacies of NETs to unlock their therapeutic potential within the cancer treatment. Unfortunately, current clinical trials have not yet defined the optimal treatment strategies for targeting NETs (159). Researchers are investigating the use of drugs to inhibit the formation of NETs. Some of these drugs can reduce NETs generation by targeting key components, such as DNA or specific proteins. Most experiments and clinical studies targeting NETs have focused on diseases other than cancer, such as autoimmune diseases and respiratory conditions or their complications (25). In autoimmune diseases like systemic lupus erythematosus, DNase has been shown to play a crucial role in degrading NETs. This mechanism allows DNase to not only reduce the number of NETs but also improve the clinical symptoms of these conditions (160). Therefore, considering that DNase can disrupt the structure of NETs, these structures could be an ideal target for DNase therapy, potentially reducing their tumor-promoting effects. In a mouse model of breast cancer, researchers applied DNase treatment. The results indicated that DNase treatment significantly reduced tumor burden, suggesting that it may inhibit tumor growth by decreasing the quantity and function of NETs (161). In a mouse model of metastatic lung cancer, systemic administration of DNase also led to a reduction in experimental metastasis (99). Although DNase performs well in experimental models, its clinical application faces many challenges. Further researches are needed to determine how to combine DNase with existing cancer treatments, such as chemotherapy and immune checkpoint inhibitors, to maximize its therapeutic effects. In another study, researchers investigated the formation of NETs induced by Bacillus Calmette-Guérin (BCG) stimulation. The results showed that BCG-induced NETs possess cytotoxic properties, capable of inducing apoptosis and cell cycle arrest, while inhibiting the migration of bladder tumor cells. Additionally, NETs play a role *in vivo* by facilitating the recruitment of T cells and monocyte-macrophages, as well as causing tissue damage, which helps prevent tumor (162). In summary, NETs can exhibit different effects under various pathological conditions, potentially promoting or inhibiting tumor growth, which complicates targeted therapy. Another important consideration is the study of biomarkers associated with NETs to determine which patients might benefit the most from targeted NETs therapy. Currently known NETs formation biomarkers, such as H3Cit and MPO-DNA, may hold prognostic value for patients with cancer (99, 163). Understanding the mechanisms that regulate neutrophil and NETs behavior in the TME creates opportunities for therapeutic interventions to reshape immune responses against tumors. By targeting pathways and factors related to neutrophil-mediated immune suppression, it may improve the effectiveness of current anti-cancer treatments and pave the way for new therapies.

## 6 Conclusions

In conclusion, the interaction between NETs and TIME plays a critical role in the progression of gynecologic cancers. Neutrophils, as a key component of the innate immune system, have been shown to directly impact tumor formation and development beyond their role in chronic inflammation. The formation of NETs can promote tumor metastasis by enhancing invasion, migration, and creating an inflammatory environment that supports tumor cell survival and proliferation. Furthermore, NETs released under stress during surgical procedures have been linked to tumor recurrence and metastasis, highlighting the complex interplay between the immune system and cancer progression.

Understanding the mechanisms by which NETs influence the TIME in gynecologic cancers is crucial for developing targeted therapies that can modulate the immune response to tumors. Further research into the specific interactions between NETs, immune cells, and tumor cells may provide insights into novel treatment strategies that can improve outcomes for patients with gynecologic cancers. By unraveling the complex dynamics of NETs in the tumor microenvironment, we can pave the way for more effective and personalized approaches to cancer therapy in the future.
